# Effects of Rehmannia glutinosa polysaccharides on bone tissue structure and skeletal muscle atrophy in rats with disuse

**DOI:** 10.1590/ACB360403

**Published:** 2021-05-14

**Authors:** Li Ou, Wenqian Kang, Jiahao Zhang, Ziyi Liang, Min Li, Feng Gao, Lin Chen

**Affiliations:** 1 PhD, Full Professor. Shaanxi University of Chinese Medicine – College of Pharmacy – Department of Clinical Chinese Pharmacy – Xianyang, China.; 2 Graduate student. Shaanxi University of Chinese Medicine – College of Pharmacy – Department of Clinical Chinese Pharmacy – Xianyang, China.; 3 PhD, Associate Professor. Shaanxi University of Chinese Medicine – College of Pharmacy – Department of Clinical Chinese Pharmacy – Xianyang, China.

**Keywords:** Herb, Ovariectomize, Ubiquitin, Oxidative Stress, Pathway

## Abstract

**Purpose:**

To study effects of Rehmannia glutinosa polysaccharides (RGP) on bone tissue structure and skeletal muscle atrophy in rats with disuse.

**Methods:**

A rat model of disuse osteoporosis combined with muscle atrophy was established by removing the bilateral ovaries of rats and fixing their hind limbs for a long time. Forty SD rats were administered intragastrically for 12 weeks. The bone histomorphometry parameters and the level of oxidative stress were measured. In addition, the changes of muscle atrophy F-box (MAFbx), muscle RING-finger protein-1 (MuRF1), forkhead box O1 (FOXO1) mRNA expression in skeletal muscle of rats were observed.

**Results:**

RGP significantly increased the percentage of fluorescence perimeter and bone mineralization deposition rate of the second lumbar vertebrae of rats. It also significantly increased the wet weight ratio and muscle fiber cross-sectional area of the gastrocnemius muscle of rats. At the same time, RGP significantly increased the levels of super oxide dismutase (SOD) and catalase (CAT) in the skeletal muscle of rats, and reduced the content of malondialdehyde (MDA). Rehmannia glutinosa polysaccharides also significantly reduced the expression levels of FOXO1, MAFbx and MuRF1 mRNA in rat skeletal muscle.

**Conclusions:**

RGP could improve the bone structure of osteoporotic rats. It could also improve muscle that atrophy may be related to the inhibition of FOXO1-mediated ubiquitin-proteasome pathway.

## Introduction

Osteoporosis is a systemic metabolic bone disease that can occur in different genders and ages, but it is more common in postmenopausal women and elderly men. Its main characteristics are bone loss and degenerative changes in bone microstructure^1^. Due to the aging population, osteoporosis has become a global health problem. According to the survey, 140 million people in China suffer from osteoporosis, and about 210 million people have bone mass below normal^2^^,^ 3. Biomechanical studies have shown that muscle loss in disuse osteoporosis is closely related to bone loss. Muscle tissue atrophy can lead to a decrease in bone mass and muscle strength, leading to falls and fractures^4^^–^ 6.

At present, the main clinical drugs for treating osteoporosis include calcium, vitamin D and bone resorption inhibitors. However, long-term use of these drugs will increase the incidence of osteosarcoma and venous thrombosis, and may cause kidney damage^7^^,^ 8. Therefore, more effective and safer intervention strategies are needed for osteoporosis treatment. Chinese herbal medicine has a long history of preventing and treating osteoporosis, and it has the advantages of definite curative effect and small side effects^9^^,^ 10. *Rehmannia glutinosa* is an herb commonly used clinically to prevent and treat osteoporosis. Studies have found that preparations of *Rehmannia glutinosa* possesses skeletal muscle protection via reducing oxidative damage and regulating protein synthesis and degradation pathways in methylglyoxal-induced atrophy of C2C12 myotubes^11^. *Rehmannia glutinosa* may help increase fat-oxidation through the induction of plasma membrane fatty acid-binding protein (FABPpm), a muscle specific transporter, in ovariectomized rat skeletal muscles^12^. *Rehmannia glutinosa* can also treat osteoporosis by inhibiting the number and differentiation of osteoblasts^13^. *Rehmannia glutinosa* polysaccharide (RGP) is the main component of *Rehmannia glutinosa*, which can enhance immunity and promote the differentiation of bone marrow mesenchymal stem cells^14^^–^ 17, but its mechanism is still unclear.

In this study, a rat model of disuse osteoporosis combined with muscle atrophy was established by removing both ovaries and immobilizing the hind limbs for a long time. The effects of RGP on bone tissue structure and skeletal muscle atrophy in rats was studied, and the mechanism of preventing and treating osteoporosis was explored.

## Methods

This study was approved by the Animal Ethics Committee of Shaanxi University of Traditional Chinese Medicine and conducted following the principles of the Care and Use of Laboratory Animal.

### Drug and reagent

*Rehmannia glutinosa* polysaccharides was purchased from Shanghai Ronghe Pharmaceutical Technology Development Co., Ltd., with UV > 95%. Alendronate sodium tablets are produced by Ouyi Pharmaceutical Co., Ltd. Sodium pentobarbital is produced by American Sigma Company. Superoxide dismutase (SOD), catalase (CAT), and malondialdehyde (MDA) kits were all provided by Nanjing Jiancheng Bioengineering Institute. The muscle atrophy F-box (MAFbx), muscle RING-finger protein-1 (MuRF1), forkhead box O1 (FOXO1) primers were provided by Nanjing GenScript Biotechnology Co., Ltd. The RNA extraction kit was provided by Beijing TransGen Biotechnology Co., Ltd.

### Ovariectomy and animal grouping

Forty SD female rats weighing 200–220 g were provided by Chengdu Dashuo Experimental Animal Co., Ltd., and the certificate number was SCXK (chuan) 2015-30. The rats were raised in separate cages and fed with standard feed. The room temperature was 18~25 ^o^C, and the relative humidity was 40~70%.

The rats were randomly divided into sham operation group, model group, positive control group and RGP group,with 10 rats in each group. Rats in the positive control group were given alendronate sodium 0.9 mg·kg^–1^ by gavage, while rats in the RGP group were given RGP400 mg·kg^–1^ by gavage every day. The sham operation group and the model group were given an equal volume of normal saline by gavage every day for 12 weeks. Except for the sham operation group, the rats in the other groups were anesthetized with sodium pentobarbital and fixed in the prone position. After shaving and disinfection, the skin and muscles were cut longitudinally along the 1 cm on both sides of the first thoracolumbar vertebrae, and the ovaries on both sides were completely removed^18^. Rats in the sham operation group were only operated on, but the ovaries were not removed. The rats were injected intramuscularly with 400,000 U·kg^–1^ of penicillin once a day after surgery for 3 days. On the 7th day after operation, the rats were immobilized by the modified Bobath method^19^. After the rats were anesthetized, three layers of cotton gauze were placed on the outer side of their left hind limbs, and then the joints of the rats were wrapped with a plaster bandage to fix their hind limbs in a shortened and bent position.

### Determination of static and dynamic bone histomorphometry parameters

After the animals were sacrificed, the second lumbar vertebral body was cut with a low-speed saw, fixed in formalin buffer for 24 h, and dehydrated with ethanol before embedding in nondecalcified bone. The embedding block was cut into two thicknesses of 5 and 10 μm with a hard tissue microtome. The 5 μm sections were stained with bone dye to measure static bone histomorphometry parameters. The 10 μm slices are directly transparent and then mounted to measure dynamic bone histomorphometry parameters. Static parameters include trabecular bone area (Tb.Ar, mm^2^), percentage of trabecular bone area (%Tb.Ar, %), trabecular bone width (Tb.Th, μm), and number of trabecular bone (Tb.N, n·mm^–2^), trabecular bone separation (Tb.Sp, μm). Dynamic parameters include fluorescence perimeter percentage (%L.Pm, %), bone surface area bone formation rate (BFR/BS, μm·d^–1^ × 100), bone tissue volume bone formation rate (BFR/TV, %·year^–1^), bone mineralization deposition rate (MAR, μm·d^–1^), number of osteoclasts per millimeter (Oc.N, N·mm^–1^).

### Determination of the wet weight ratio of gastrocnemius tissues and the cross-sectional area of muscle fibers

The gastrocnemius muscles on both sides of the rat were stripped and weighed, and the wet weight ratio of the gastrocnemius muscle tissues on both sides of the rat was calculated. After staining with hematoxylin and eosin, the cross-sectional area of muscle fibers was measured using ImageJ software.

### Determination of oxidative stress level in rat skeletal muscle

After the stripped left gastrocnemius muscle was washed with PBS, it was fully homogenized in a homogenizer, centrifuged at 2500 r·min^–1^ for 10 min, and the supernatant was taken for detection of SOD, CAT, and MDA content.

### Real-time quantitative PCR detection of MAFbx, MuRF1, FOXO1 mRNA expression

The stripped left soleus muscle of the rat was pulverized in a mortar with liquid nitrogen, and the corresponding volume of trizol was added to extract RNA. It was amplified according to the set conditions after reverse transcription. Reaction conditions: 95 ^o^C 5 min, 95 ^o^C 30 s, 59 ^o^C 30 s, 72 ^o^C 30 s. The results were analyzed by the –2^-ΔΔct^ method after 40 cycles. The primers of MAFbx, MuRF1 and FOXO1 were designed by primer design software (Table 1).

**Table 1:** Primers used for PCR.

Gene	Primers	Primer length (bp)
MAFbx	F: 5´-TCCTGGATTCCAGAAGATTCAAC-3´	75
	R: 5´-TCAGGGATGTGAGCTGTGACTT-3´	
MuRF1	R: 5´-GTGCCAACGACATCTTCCAG-3´	158
	F: 5´-TTCCACCAGCAGGTTCCTCT-3´ GCTGCACGCGGAGCTGCGTGAAA-3´	
FOXO1	R: 5´-AGGCCTCCCAGGACTACAGA-3´	341
	F: 5´-ACAGGTATTTGGGGCAGCAT-3´ GCTGCACGCGGAGCTGCGTGAAA-3´	

### Statistical analysis

The experimental data were expressed as mean ± standard deviation (χ ± s) and statistically analyzed by SPSS19.0 software. Differences between the two groups were analyzed for significance by t-test. P-values of 0.05 or less were regarded as statistically significant.

## Results

### Effects on changes in bone structure of rats

**Figure 1:**
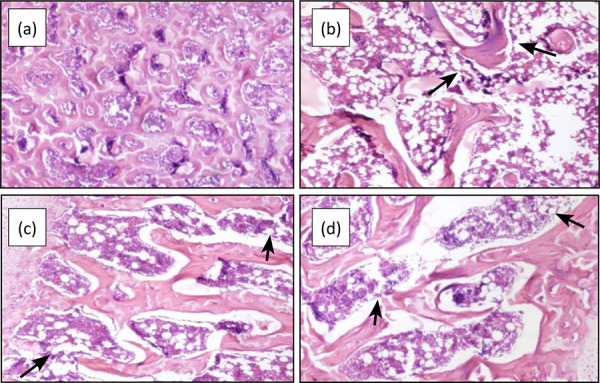
Effect on changes in bone structure of rats (HE 40×). **(a)** Sham operation group; **(b)** Model group; **(c)** Positive control group; **(d)** RGP group.

The results of hematoxylin-eosin staining (HE) staining showed that the trabeculae of the sham-operated rats were arranged regularly and tightly, the periosteum was intact, and there were abundant osteoblasts in the medullary cavity (Fig. 1a). The trabecular bones of the rats in the model group were arranged disorderly and the distance between trabecular bones was enlarged, and there were large areas of trabecular bone loss (Fig. 1b). Compared with the model group, the trabecular bones of rats in the positive control group and RGP group were arranged more regularly. A few trabeculae of bones were broken (Fig. 1c), and the periosteum was relatively intact (Fig. 1d).

### Effect on static bone histomorphometry parameters

**Figure 2:**
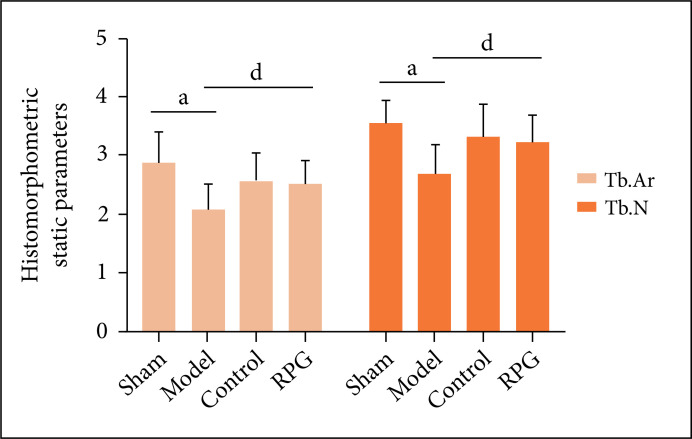
Effects on Tb.Ar and Tb.N. ^a^p < 0.01 compared with sham group; ^d^p < 0.05 compared with model group.

**Figure 3:**
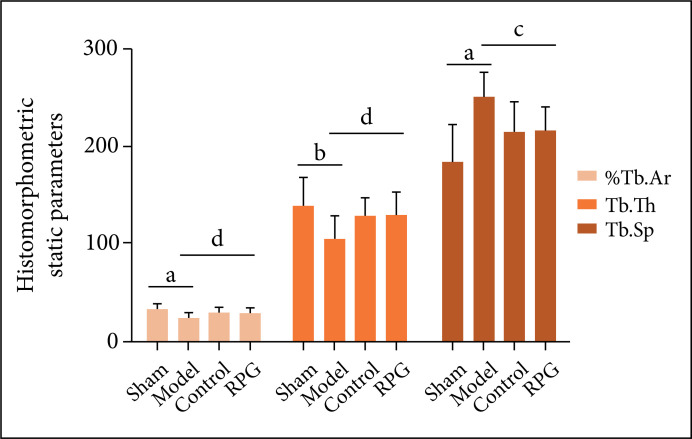
Effects on %Tb.Ar, Tb.Th and Tb.Sp. ^a^p < 0.01, ^b^p < 0.05 compared with sham group; ^c^p < 0.01, ^d^p < 0.05 compared with model group.

Compared with the sham operation group, Tb.Ar, %Tb.Ar and Tb.N of the model group were significantly decreased (p < 0.01), Tb.Th (p < 0.05) was significantly decreased, and Tb.Sp was significantly increased (p < 0.01). Compared with the model group, %Tb.Ar, Tb.N and Tb.Th of rats in the RGP group were significantly increased (p < 0.05), and Tb.Sp was significantly reduced (p < 0.01, Figs. 2 and 3).

### Effect on dynamic bone histomorphometry parameters

**Figure 4:**
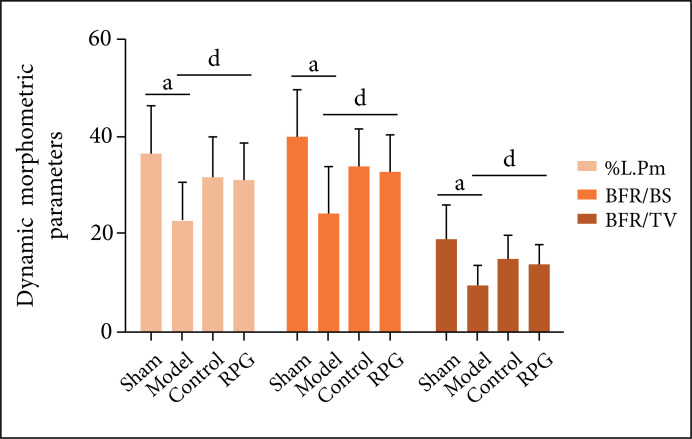
Effects on %L.Pm, BFR/BS and BFR/TV. ^a^p < 0.01 compared with sham group; ^d^p < 0.05 compared with model group.

**Figure 5:**
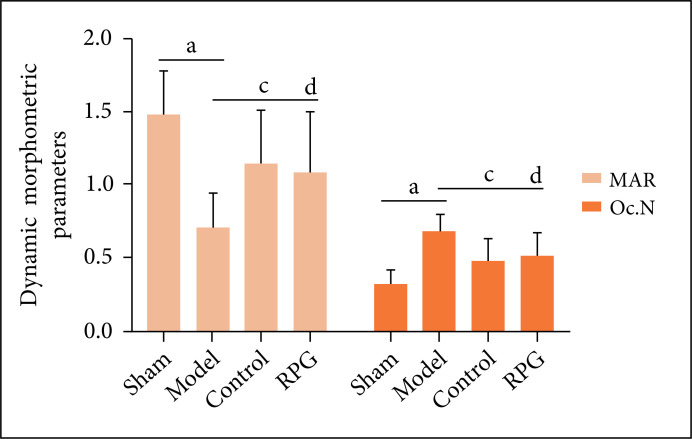
Effects on MAR and Oc.N. ^a^p < 0.01 compared with sham group; ^c^p < 0.01, ^d^p < 0.05 compared with model group.

Compared with the sham operation group, %L.Pm, BFR/TV and MAR of the model group were significantly reduced (p < 0.01), and Oc.N was significantly increased (p < 0.01). Compared with the model group, %L.Pm, BFR/TV and MAR of the RGP group were significantly increased (p < 0.05), while Oc.N was significantly decreased (p < 0.05, Figs. 4 and 5).

### Effect on wet weight ratio and cross-sectional area

**Figure 6:**
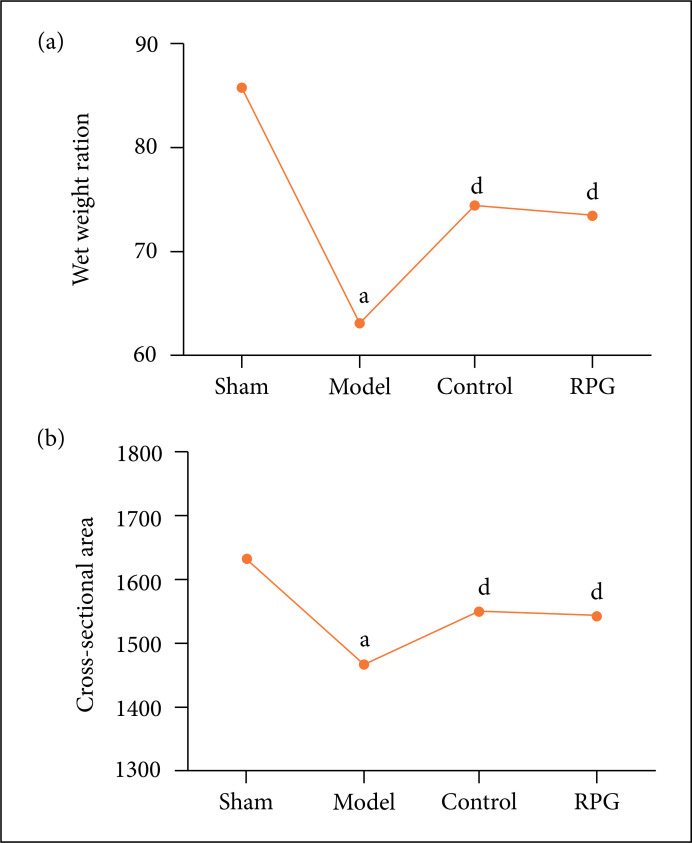
Effect on wet weight ratio and cross-sectional area in rat gastrocnemius muscle. **(a)** Effect on wet weight ratio; **(b)** Effect on cross-sectional area. ^a^P < 0.01 compared with sham group; ^d^p < 0.05 compared with model group.

Compared with other groups, rats in the sham operation group had the highest gastrocnemius wet weight ratio and the largest cross-sectional area of muscle fibers. Compared with the sham operation group, the wet weight ratio and the cross-sectional area of muscle fibers of the model group, alendronate sodium group and RGP group were significantly reduced, and the wet weight ratio of the model group decreased more significantly (p < 0.01). Compared with the model group, the wet weight ratio of gastrocnemius and the cross-sectional area of muscle fibers in the RGP group were significantly increased (p < 0.05, Fig. 6), and local muscle atrophy was improved.

### Effect on the oxidative stress in rat gastrocnemius muscle

Compared with the sham operation group, the SOD and CAT contents in the skeletal muscle tissue of the model group were significantly reduced (p < 0.01), while the MDA content was significantly increased (p < 0.01). Compared with the model group, the SOD and CAT contents of the skeletal muscle of the RGP group were significantly increased, and the difference was statistically significant (p < 0.05), while the MDA content was significantly decreased (p < 0.05, Table 2).

**Table 2:** Effect on the oxidative stress in rat gastrocnemius muscle (*x* ± s, n = 10).

Group	SOD	CAT	MDA
Sham	11.38 ± 2.54	16.23 ± 2.97	3.18 ± 0.76
Model	6.63 ± 1.29^a^	12.47 ± 2.85^c^	5.46 ± 0.84^a^
Control	8.85 ± 2.03^c^	14.89 ± 2.12^d^	4.20 ± 1.33^d^
RGP	8.93 ± 2.11^c^	14.95 ± 2.20^d^	4.24 ± 1.21^d^

a p < 0.01,

b p < 0.05, compared with sham operation group.

c p < 0.01,

d p < 0.05, compared with model group.

### Effects on MAFbx, MuRF1, FOXO1 mRNA expression

**Figure 7:**
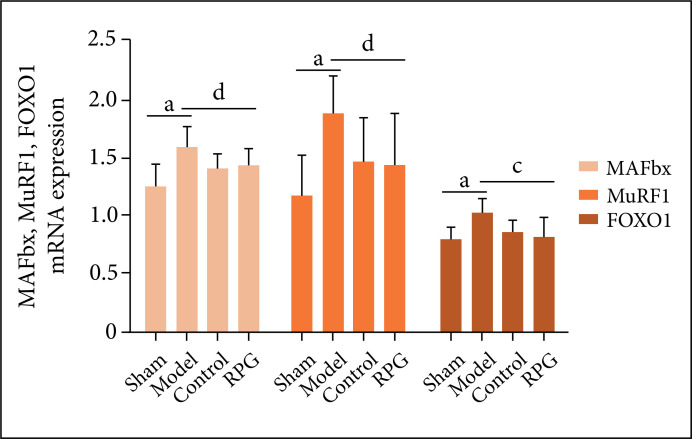
Effects on MAFbx, MuRF1, FOXO1 mRNA expression. ^a^p < 0.01 compared with sham group. ^c^p < 0.01, ^d^p < 0.05 compared with model group.

Compared with the sham operation group, the gene expression of MAFbx, MuRF1 and FOXO1 in the bone tissues of the other groups was significantly increased (p < 0.01). Compared with the model group, the FOXO1 gene expression in the bone tissue of the RGP group was significantly reduced (p < 0.01), and the gene expression of MAFbx and MuRF1 was also significantly reduced (p < 0.05, Fig. 7).

## Discussion

The development, function and aging of the skeletal and muscular system are an organic whole, and the formation of osteoporosis is closely related to the decline in coordination of bones and skeletal muscles. In addition to mechanical coupling between bone and muscle, there is also a coexistence and adaptation relationship in biosynthesis. For example, the bone mass of the bones will continue to increase the mass of the muscles before reaching the peak, and the loss of bone mass will be accompanied by muscle atrophy^20^.

When rats are ovariectomized and their limbs are fixed for a long time, due to the decrease of estrogen level and lack of mechanical stress stimulation, the unloading of skeletal muscle and bone tissue will undergo a series of physiological, structural and functional changes. The balance between bone formation and bone resorption is broken, resulting in increased osteoclast-mediated bone resorption with less bone formation. At the same time, the anabolism of muscle protein is decreased, and the slow-twitch fibers are converted to fast-twitch fibers, which causes the reduction of systemic or local bone mass and muscle mass, and ultimately leads to the formation of disuse osteoporosis and muscle atrophy. In this study, a rat model of disuse osteoporosis combined with muscle atrophy was established by removing the bilateral ovaries of rats and fixing their hind limbs for a long time.

The US Food and Drug Administration takes bone histomorphometric parameters as one of the pharmacodynamic standards for evaluating new drugs for the treatment of osteoporosis^21^. The experimental results showed that the static and dynamic bone histomorphometry parameters of the second lumbar vertebrae of the rats in the model group changed significantly after modeling, among which Tb.Ar, %Tb.Ar, Tb.N, %L.Pm, BFR/TV and MAR both decreased significantly, Tb.Th decreased significantly, Tb.Sp and Oc.N increased significantly, and the wet weight ratio of muscle and the cross-sectional area of muscle fibers decreased significantly, proving the successful establishment of the animal model.

The results of this experiment showed that RGP could significantly increase %Tb.Ar, Tb.N and Tb.Th, and significantly reduce Tb.Sp of ovariectomized rats. The measurement results of dynamic bone histomorphometry parameters showed that RGP could significantly increase the %L.Pm, BFR/TV and MAR, and significantly reduce Oc.N of ovariectomized rats. The results of this study confirmed that RGP could promote bone formation and inhibit bone resorption, so that the bone structure was improved, which had a certain therapeutic effect on osteoporosis in ovariectomized rats. *Rehmannia glutinosa* polysaccharides could also significantly increase the wet weight ratio and muscle fiber cross-sectional area of the gastrocnemius muscle of disuse osteoporotic rats, and improve the state of local muscle atrophy in rats.

Studies have shown that oxidative stress caused by reactive oxygen free radicals is an important cause of disuse muscle atrophy. When a large number of free radicals appear in skeletal muscle, they promote lipid peroxidation of macromolecular cells, and the decline of mitochondrial function causes energy deficiency, which causes muscle dysplasia and damage^22^^–^ 25. Superoxide dismutase and CAT are important antioxidant enzymes that can scavenge oxygen free radicals. Malondialdehyde is a lipid peroxidation product produced in the metabolism of oxygen free radicals, and its content can reflect the level of lipid peroxidation. The results of the study showed that RGP significantly increased the content of SOD and CAT in rat skeletal muscle, and decreased the content of MDA, indicating that RGP could improve the oxidative stress state of rat skeletal muscle after long-term limb fixation.

Oxidative stress mainly leads to a decrease in skeletal muscle mass by increasing protein hydrolysis. Under oxidative stress, the production of oxygen free radicals will increase protein degradation, reduce protein synthesis, and affect DNA repair^26^. Oxidative stress induced activation of the ubiquitin-proteasome pathway is an important cause of skeletal muscle atrophy. The ubiquitin-proteasome system can degrade misfolded or unfolded proteins and is the main way of protein degradation in cells. The MAFbx and MuRF1 are the two most common muscle-specific E3 ubiquitin-protein ligases. They regulate ubiquitin-mediated protein degradation in skeletal muscle and can be used as early molecular markers of skeletal muscle atrophy^27^^,^ 28. Insufficient secretion of hormones caused by aging will cause the level of IGF-1 to decrease, and FOXO will return to the nucleus after dephosphorylation. This induces the expression of muscle-specific ubiquitin protein ligases MuRF1 and MAFbx, resulting in decreased muscle synthesis and muscle atrophy. The results of this study showed that RGP significantly reduced the expression levels of FOXO1, MAFbx and MuRF1 mRNA in rat skeletal muscle, indicating that its mechanism of improving muscle atrophy may be related to the inhibition of FOXO1-mediated ubiquitin-proteasome pathway.

## Conclusion

*Rehmannia glutinosa* polysaccharides could promote bone formation and inhibit bone resorption to improve the bone structure of osteoporotic rats. *Rehmannia glutinosa* polysaccharides could also improve muscle atrophy and may be related to the inhibition of FOXO1-mediated ubiquitin-proteasome pathway.
